# Mapping the continuous reciprocal space intensity distribution of X-ray serial crystallography

**DOI:** 10.1098/rstb.2013.0333

**Published:** 2014-07-17

**Authors:** Oleksandr Yefanov, Cornelius Gati, Gleb Bourenkov, Richard A. Kirian, Thomas A. White, John C. H. Spence, Henry N. Chapman, Anton Barty

**Affiliations:** 1 Center for Free-Electron Laser Science, Deutches Elektronen-Synchrotron DESY, Notkestrasse 85, 22607 Hamburg, Germany; 2 European Molecular Biology Laboratory, Hamburg, Germany; 3 Department of Physics, Arizona State University, Tempe, AZ 85287, USA; 4 Department of Physics, University of Hamburg, Luruper Chaussee 149, Hamburg 22607, Germany; 5 Center for Ultrafast Imaging, Luruper Chaussee 149, Hamburg 22761, Germany

**Keywords:** serial crystallography, free-electron laser, three-dimensional diffraction

## Abstract

Serial crystallography using X-ray free-electron lasers enables the collection of tens of thousands of measurements from an equal number of individual crystals, each of which can be smaller than 1 µm in size. This manuscript describes an alternative way of handling diffraction data recorded by serial femtosecond crystallography, by mapping the diffracted intensities into three-dimensional reciprocal space rather than integrating each image in two dimensions as in the classical approach. We call this procedure ‘three-dimensional merging’. This procedure retains information about asymmetry in Bragg peaks and diffracted intensities between Bragg spots. This intensity distribution can be used to extract reflection intensities for structure determination and opens up novel avenues for post-refinement, while observed intensity between Bragg peaks and peak asymmetry are of potential use in novel direct phasing strategies.

## 1. Introduction

X-ray free-electron lasers have opened up new avenues of structure determination by using intense X-ray pulses of sufficiently short duration to outrun most conventional radiation damage processes [1–4]. In particular, the technique of serial femtosecond crystallography (SFX) enables the study at room temperature of crystals that are either too small or too radiation sensitive to be studied using traditional X-ray crystallography approaches [1,5–7]. In serial crystallography, individual crystals are introduced one after another into the X-ray beam and exposed to a single X-ray pulse of several femtoseconds duration. Each exposure records the diffraction pattern from a separate crystal in a random orientation, while femtosecond exposures effectively freeze any crystal rotation during exposure. The ability to operate at room temperature without the need for cryogenic conditions opens up new possibilities in time-resolved crystallography [8,9].

Current data analysis methods for serial crystallography produce intensities for structure determination by indexing the diffraction pattern from each crystal separately, locally integrating the intensity of every reflection predicted to be present in the diffraction pattern and merging reflection intensities from many thousands of crystals [7,10–12]. The final set of merged reflection intensities represents an average across all measured crystals. Problems of crystal variability, partial intersection of reflections with the Ewald sphere in each individual exposure and spectral fluctuations are addressed by summing many separate measurements of each reflection obtained from separate exposures using Monte Carlo integration [7,12]. Information about the intensity distribution around and between Bragg peaks is discarded at this indexing-and-integration step.

The use of small crystals and highly coherent X-ray beams has sparked interest in exploiting intensity information *between* Bragg peaks for use in structure determination [10,11,13,14]. This is similar to the approach of single-particle imaging, where samples are not crystalline and the reciprocal space intensity distribution is a continuous function. Diffraction patterns from identical non-crystalline objects are oriented and assembled in three dimensions to recover the full three-dimensional reciprocal space intensity distribution [15–18], from which the structure can be determined using phase retrieval techniques [2]. By design, these techniques preserve the whole three-dimensional reciprocal space intensity distribution.

Here, we apply techniques derived from the field of single-particle imaging to the analysis of serial crystallography data. We first perform a ‘three-dimensional merge’ of the data from nanocrystals of different sizes, by placing diffraction patterns from individual crystals in three-dimensional space using crystal orientation information determined by autoindexing routines [7,19,20]. Refinement and scaling of diffraction pattern orientation can be performed using orientation determination algorithms borrowed from single-particle imaging [15–18,21]. Intensities extracted from the three-dimensional reciprocal space intensity distribution are suitable for conventional crystallographic analysis, while information about peak asymmetry and intensity between reciprocal lattice points is preserved for use in direct-phasing strategies [10,11,13,14,22].

## 2. Assembly of diffraction patterns in three dimensions

**Figure 1.**
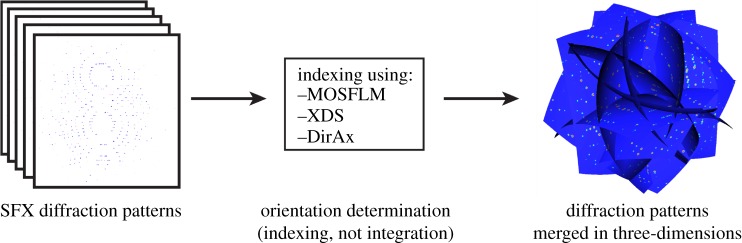
In three-dimensional merge, individual diffraction patterns are indexed to determine the orientation of each individual crystal, and the orientation information used to assemble diffraction patterns in three-dimensional reciprocal space. There is no integration of intensities or profile fitting performed at the indexing step; rather the continuous three-dimensional reciprocal space intensity distribution is obtained by averaging of all diffraction patterns in three dimensions. Reflection intensities are extracted after merging in three dimensions. (Online version in colour.)

The three-dimensional merge principle is illustrated in figure 1. Conventional autoindexing routines applied to individual diffraction patterns provide the crystal reciprocal lattice in the laboratory frame and the orientation information from this lattice is used to assemble diffraction patterns in three-dimensional reciprocal space. Assembly of multiple diffraction patterns in this way produces a continuous three-dimensional intensity distribution that reflects the average over all merged crystals. This three-dimensional assembly procedure is very similar to the procedure used for structure determination in three-dimensional diffractive imaging [2,23], except that for SFX data the sample is crystalline, and orientation is determined using crystal indexing software rather than a goniometer. For SFX data, the resultant three-dimensional reciprocal space intensity distribution contains Bragg peaks from the crystal reciprocal lattice, including any asymmetry related to the underlying molecular transform. We call this process ‘three-dimensional merging’ to avoid confusion with the term ‘three-dimensional refinement’ used in crystallography to refer to existing profile fitting approaches [20,24], even though orientation and scaling refinement can be performed as a part of the three-dimensional merge.

We used the published dataset of cathepsin B [1] for testing the three-dimensional merge approach. For that dataset, crystals of cathepsin B were grown *in vivo*, producing long needles with an average length of 11 µm and square cross-section of 0.5–1.0 µm on a side [1]. Crystals were injected at room temperature as a liquid suspension using a gas dynamic virtual nozzle and coherently illuminated by an X-ray beam of 4 µm diameter in the CXI instrument [25] at the Linac Coherent Light Source (LCLS) at a photon energy of 9.4 keV (1.3 Å wavelength). Nearly 4 million individual data frames were collected. Crystal unit cell dimensions were determined by indexing to be *a* = *b* = 125.4 Å, *c* = 54.6 Å with space group P4_2_2_1_2. In the original analysis [1], 293 195 events contained useful diffraction from which 178 875 (61%) diffraction patterns could be indexed and integrated into structure factors using the CrystFEL analysis pipeline [7].

Reprocessing the dataset using a more recent version of CrystFEL (0.5.1) and an improved detector geometry specification resulted in 209 866 indexable diffraction patterns. Merging of the cathepsin B data in three dimensions was performed by first subtracting a radially symmetric photon background from each diffraction pattern, followed by mapping diffraction pattern intensities onto the Ewald sphere defined by the wavelength and detector geometry of the diffraction pattern. The reciprocal lattice vectors obtained from indexing of each pattern were used to determine the orientation of the Ewald sphere in the frame of the crystal lattice in order to insert each diffraction pattern into a three-dimensional array. Effects of spectral bandwidth and finite beam convergence were ignored for the time being even though they could have been taken into account in this summation procedure by distributing intensities according to a weighting corresponding to a spectrum measured for each shot (in our case, only an estimated average spectrum was known). We instead assumed a thin Ewald sphere with summation onto the regular three-dimensional grid performed using a weighted sum defined by voxel overlap. Finally, known symmetry operations for the crystal point group were applied to the data as a further averaging step.

**Figure 2.**
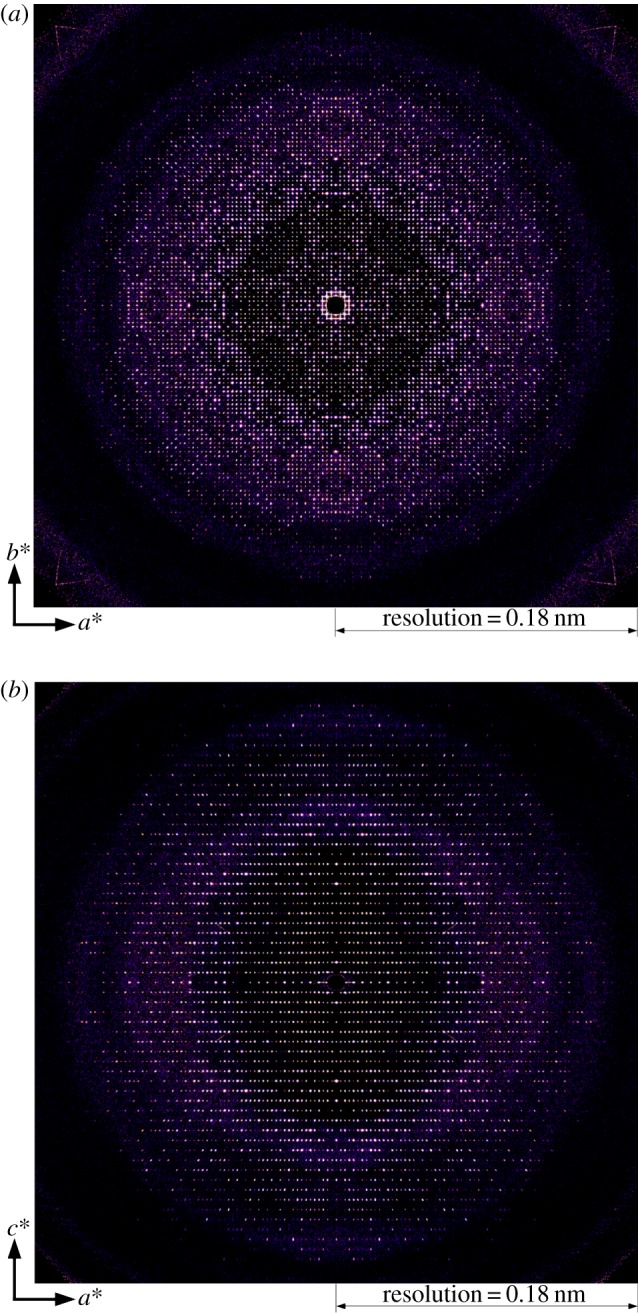
Cuts through the continuous three-dimensional reciprocal space intensity distribution for experimental cathepsin B data assembled from 209 866 individual femtosecond diffraction snapshots measured at the LCLS. (*a*) Cut through the h–k (*l* = 0) plane (a*/b* direction), and (*b*) orthogonal cut through the h–l (*k* = 0) plane (a*/c* direction). The point group symmetry 4/mmm has been applied to this data to improve averaging, consistent with the crystal space group P4_2_2_1_2. (Online version in colour.)

Slices through the reconstructed continuous reciprocal space intensity distribution are shown in figure 2. Figure 2*a* shows a cut through the hk0 plane (cut parallel to the a*/b* directions), and figure 2*b* an orthogonal cut through the h0l plane (cut parallel to the a*/c* directions). The effect of crystal size on the reciprocal lattice of Bragg reflections is clearly visible, with the shorter *c*-axis unit cell giving rise to a larger peak spacing in the c* direction as expected. Close inspection of figure 2 shows the presence of expected forbidden reflections along the a*, b* and c* directions.

**Figure 3.**
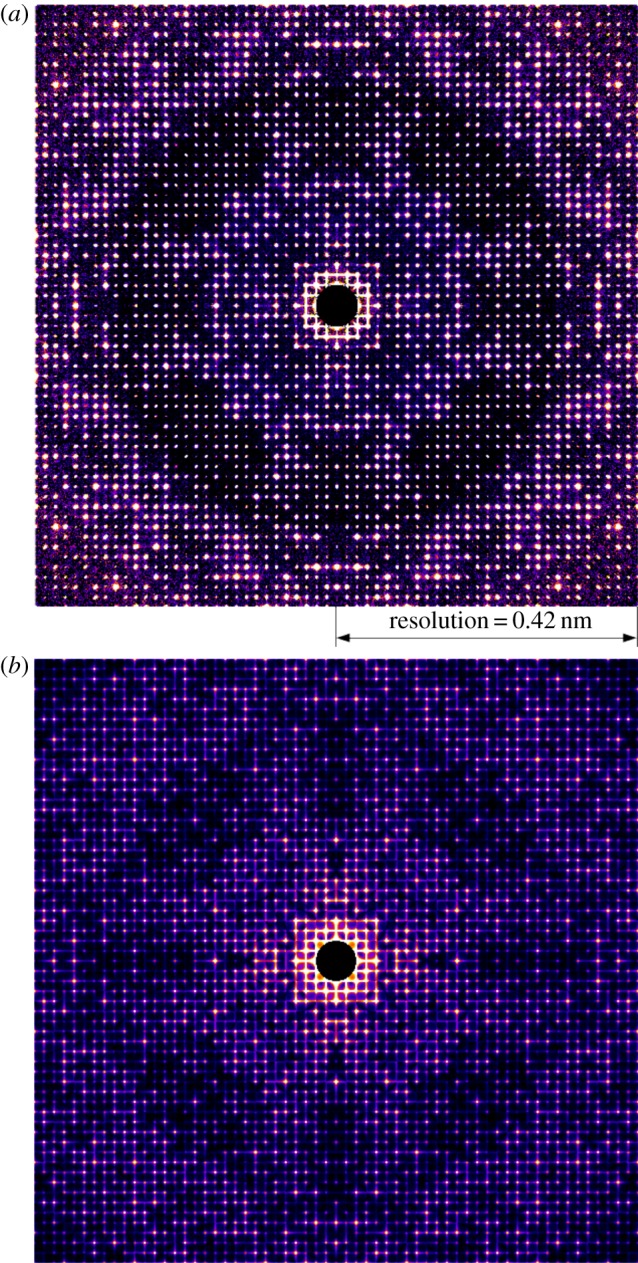
(*a*) Expanded view of a slice through the measured three-dimensional reciprocal space intensity distribution figure 1*a* showing intensity between Bragg peaks in the a*/b* direction and asymmetry in peak shape. (*b*) A simulation of the same diffraction pattern using a finite crystal consisting of 5 × 5 unit cells. (Online version in colour.)

Figure 3*a* contains an expanded view of the central portion of figure 2*a*. Of particular interest is the intensity between Bragg peaks parallel to the a*/b* reciprocal lattice directions seen in the measured reciprocal space intensity. Although the intensity between peaks is orders of magnitude weaker than the peaks themselves, it is nevertheless visible as is the resulting asymmetry in peak shape. This intensity between Bragg peaks is not visible along the c* direction. Increased intensity between Bragg peaks along a*/b* but not c* is consistent with rod-like crystals that are smaller than the illuminating X-ray beam in the a*/b* direction, whereas in the c* direction the rod-like crystals are larger than the X-ray beam size. Individual cathepsin B crystals are approximately 0.9 µm square cross-section and have sharply defined facets illuminated by the X-ray laser beam of focal spot of approximately 4 µm full width at half maximum and coherence width greater than that of the entire crystallite, unlike the normal situation with synchrotron data. No fringes are visible in the merged three-dimensional data because the detector pixel size is too large to resolve any fringes, and due to summation of data from many individual crystals of different sizes and therefore different fringe spacings. A simulated diffraction pattern of cathepsin B using finite crystals of square cross-section shows similar streaks between Bragg peaks as visible in the experimental data (figure 3*b*) supporting this hypothesis as to the origin of the streaks. Although variations in the reciprocal space intensity distribution around each reflection could be related to a range of factors including thermal diffuse scattering or anisotropic mosaicity averaged over the large number of crystals used to construct the three-dimensional reciprocal space intensity map, the similarity in intensity between Bragg peaks for both simulated and measured data suggest shape transforms as a likely cause.

**Figure 4.**
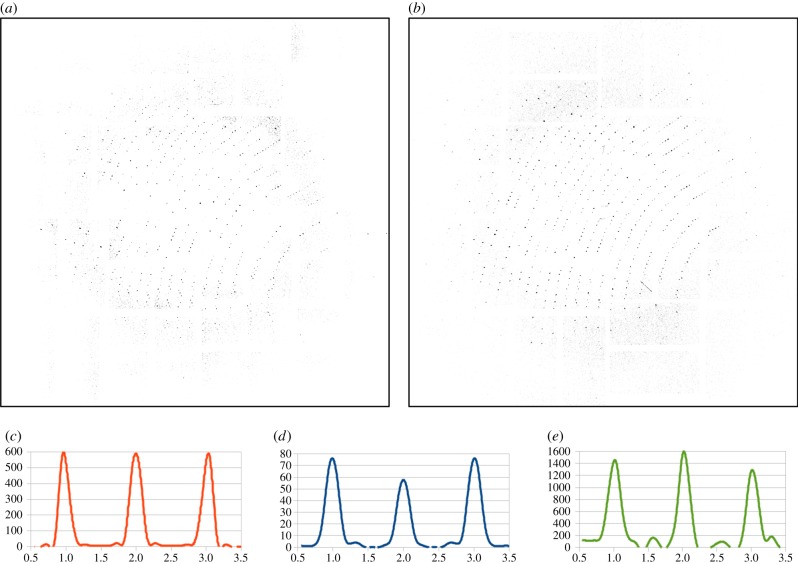
(*a*) A single-shot diffraction pattern measured at the LCLS, and (*b*) an Ewald sphere slice through the merged three-dimensional diffraction volume in the same orientation. Below are lineout sections through the reciprocal space intensity distribution of figure 2*a*. The central peak in (*c*) is the (6,0,0) reflection and (*d*) the (46,0,0) reflection, respectively. No obvious effect of peak broadening is seen for higher order reflections, indicating that orientation misalignment is minimal. Note also the presence of some asymmetry in peak profiles. A lineout from a single pattern is shown in (*e*) for the purposes of comparison. (Online version in colour.)

A single-pulse diffraction pattern measured at the LCLS is shown in figure 4*a* beside an Ewald sphere slice through the continuous reciprocal space intensity distribution in the same crystal orientation (figure 4*b*). The extracted diffraction pattern has increased signal-to-noise ratio due to averaging of multiple data frames. Missing peaks in the single-shot measurement could result from spectral fluctuation in individual SASE pulses that have been integrated over many observations in the three-dimensional merge, a significant difference in crystal shape from the average, non-uniform phase of the illuminating wavefront, or due to a slight error in the estimation of the orientation of the pattern.

Extraction of line profiles through the reciprocal space intensity distribution of figure 2*a* for both the 6,0,0 and 46,0,0 reflection along a* direction are shown in figure 4*c* and *d*, respectively. The line profiles range over the nearest neighbouring Bragg peaks. The peaks profiles are remarkably consistent in width at high and low resolution, indicating that the effects of diffraction pattern misalignment are minimal and that our assumption of the thin Ewald sphere is valid. Errors in crystal orientation determination would result in a broadening of high-order peaks relative to low-order peaks. Note also the presence of some asymmetry in peak profiles, which may reveal information about the underlying molecular transform [10]. A lineout from a single pattern is shown in (*e*) for the purposes of comparison of peak profiles in the merged data relative to what is observed in an individual diffraction measurement.

## 3. Extraction of reflection intensities for structure determination

Reflection intensities can be extracted from the three-dimensional volume for use in conventional crystallographic software. Reciprocal lattice positions are first identified in three-dimensional space and integration of intensities around each Bragg peak performed using a three-dimensional variation on the traditional three-ring background subtraction method [7] using concentric spheres for integration and background estimation. Information about intensity between Bragg peaks is discarded in this step, at which point the three-dimensional merge approach is used solely as an alternative method for the integration and merging of partial reflection data from thousands of crystals.

**Figure 5.**
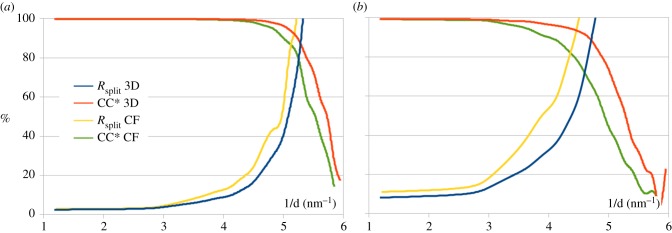
Comparison of the *R*_split_ and CC* data quality metrics as a function of resolution for both the three-dimensional merge and data merged using CrystFEL. (*a*) Symmetrized data (4/mmm) and (*b*) data without symmetry. (Online version in colour.)

The quality of three-dimensional merge data can be estimated using the *R*_split_ and CC* metrics [7,26], in which the dataset is divided into two sets of randomly selected images. A three-dimensional merge was performed independently on each half dataset, and reflection intensities extracted from each half dataset for comparison using *R*_split_ and CC*. We also re-processed the data using the most recent CrystFEL (v. 0.5.1) with an updated detector geometry for comparison with the results of three-dimensional merging. The comparison is shown in figure 5: both *R*_split_ and CC* indicate that the resolution of the three-dimensional merge data is slightly higher than that for the corresponding CrystFEL integration procedure (figure 5*a*). This is even more prominent for data merged with P1 symmetry (figure 5*b*). The atomic model of cathepsin B (PDB Code: 4HWY[1]) was refined using structure factor moduli extracted from the three-dimensional data with program REFMAC [27], with minor manual adjustments made using program COOT [28]. The resulting *R* factors are *R*/*R*_free_ = 0.176/0.211 at 2.0 Å resolution and *R*/*R*_free_ = 0.167/0.206 at 2.1 Å. This is slightly higher resolution than the original publication, 2.0 Å versus 2.1 Å. Otherwise, the structure is very consistent with results obtained using CrystFEL reflection intensities. The improvement may be due to the different method of background subtraction employed for three-dimensional merged dataset compared with the dataset processed with CrystFEL.

## 4. Discussion and ideas for future development

The three-dimensional merge approach opens up several avenues for further exploration. The first application is to the integration and refinement of datasets with partial reflections. Partial reflections occur when the Ewald sphere does not fully intersect a Bragg peak in reciprocal space [5,19,20,29–31] and have been of particular concern in SFX data processing due to the narrow spectral bandwith, low convergence angle of the incident X-ray beam and absence of crystal rotation. Partiality is addressed during the three-dimensional merge procedure by placing intensity into the three-dimensional reciprocal space voxels determined by crystal orientation and incident beam parameters. When the Ewald sphere corresponding to a particular crystal orientation fully intersects the maxima of the Bragg peak, intensity is summed into the voxel at that Bragg peak location. Conversely, when the Ewald sphere passes adjacent to the Bragg peak location intensity is placed into voxels adjacent to the Bragg peak, as determined by crytstal orientation. In order for reflections to be separated in three-dimensional reciprocal space, the excited three-dimensional reciprocal space volume must be less than the spacing between reciprocal lattice points. This requirement stems from the need to separate reflections in reciprocal space. The task of assembling data in three dimensions becomes more complicated for the case of Laue diffraction, where the use of large bandwidth gives an Ewald wedge that spans several reflections in reciprocal space. The three-dimensional intensity distribution would take on a spectral variation in a broad wavelength band case. The three-dimensional merge approach thereby benefits from the collection of a very large number of measurements with *decreased* Ewald sphere thickness through the use of narrow bandwidth illumination, limited beam convergence and no crystal rotation. Averaging data from a very large number of individual measurements in random crystal orientations is instead used to fill out reciprocal space. This contrasts to the approach typically employed in protein crystallography where increased bandwidth and/or crystal rotation are used to *increase* the effective Ewald sphere thickness in each measurement, the purpose being to decrease the number of measurements required. As such the three-dimensional merge approach may find application outside of SFX data in the processing of fine slice data from synchrotrons, for example in the merging and scaling of data collected from multiple crystals.

A second application of three-dimensional merging is the data improvement through the post-refinement of diffraction pattern orientation and scaling against the three-dimensional intensity model. To first approximation, the three-dimensional merged intensities directly provide the average partiality model and can be useful for image scaling or the resolution of indexing ambiguities. Sophisticated algorithms have been developed in the field of single-particle imaging for determining the orientation and scaling of individual diffraction patterns in three dimensions [15–18]. These algorithms could be easily adapted to the refinement of serial crystallography data. Expectation maximization methods [14,15,17] and manifold embedding [11,16] could be especially useful for refining the orientation of patterns of low signal. It may be possible to use such methods to directly determine a model for the continuous molecular transform, by implementing a shot-by-shot model for the highly symmetric reciprocal space lattice that modulates this common underlying transform. Provided the data quality in individual diffraction patterns is sufficiently high to enable successful classification or clustering of states, Bayesian methods applied to the separation of conformational states in single-particle cryo-electron microscopy [22] could be employed to sort crystals into different isomorphous classes or for the sequencing individual diffraction patterns into time steps along a reaction coordinate [32].

Finally, assembling the continuous three-dimensional reciprocal space intensity provides access to new information about the crystal structure. If all nanocrystals were identical, the three-dimensional volume around a single Bragg peak could be assembled and used for recovery of the sample shape and internal structure [33]. The requirement for all crystals to be identical is unlikely to be the case for serial crystallography data. Nevertheless, the clearly present intensity between Bragg peaks may be useful for revealing the underlying molecular transform [10] or for the extraction of intensity gradients for phasing crystal data[14]. The three-dimensional merge approach may thereby open the door to a range of novel phasing strategies [10,11,13,14,22].

## 5. Conclusion

The underlying continuous three-dimensional reciprocal space intensity distribution of an SFX dataset can be revealed by merging intensities from thousands of measurements from an equal number of individual crystals in three dimensions. The dynamic range of the data increases as more patterns are averaged, revealing the full three-dimensional reciprocal space intensity distribution, including peak asymmetry and intensities between Bragg peaks. Reflection intensities extracted from the three-dimensional volume produce slightly better *R*_split_ and CC* plots than data analysed using CrystFEL. Structures refined using intensities from three-dimensional merge are virtually indistinguishable from structures refined from CrystFEL data and produce comparable *R*_free_ values.

A key element in assembling the three-dimensional diffraction volume is the acquisition of a highly redundant still-frame dataset with thin Ewald sphere sections. Comparing individual diffraction patterns against models of the continuous reciprocal space intensity distribution enables the use of orientation determination algorithms originally devised for non-crystalline particles to be adapted for the purpose of serial crystallography data analysis, providing a new path towards orientation post-refinement, intensity pattern scaling and the resolution of indexing ambiguities. Our approach opens up new avenues for the analysis of ‘still-frame’ crystallography data using large datasets. The techniques presented here are equally applicable to the analysis of synchrotron datasets, in particular the merging of shutter-less fine slice data collected from multiple crystals and synchrotron-based serial crystallography.
